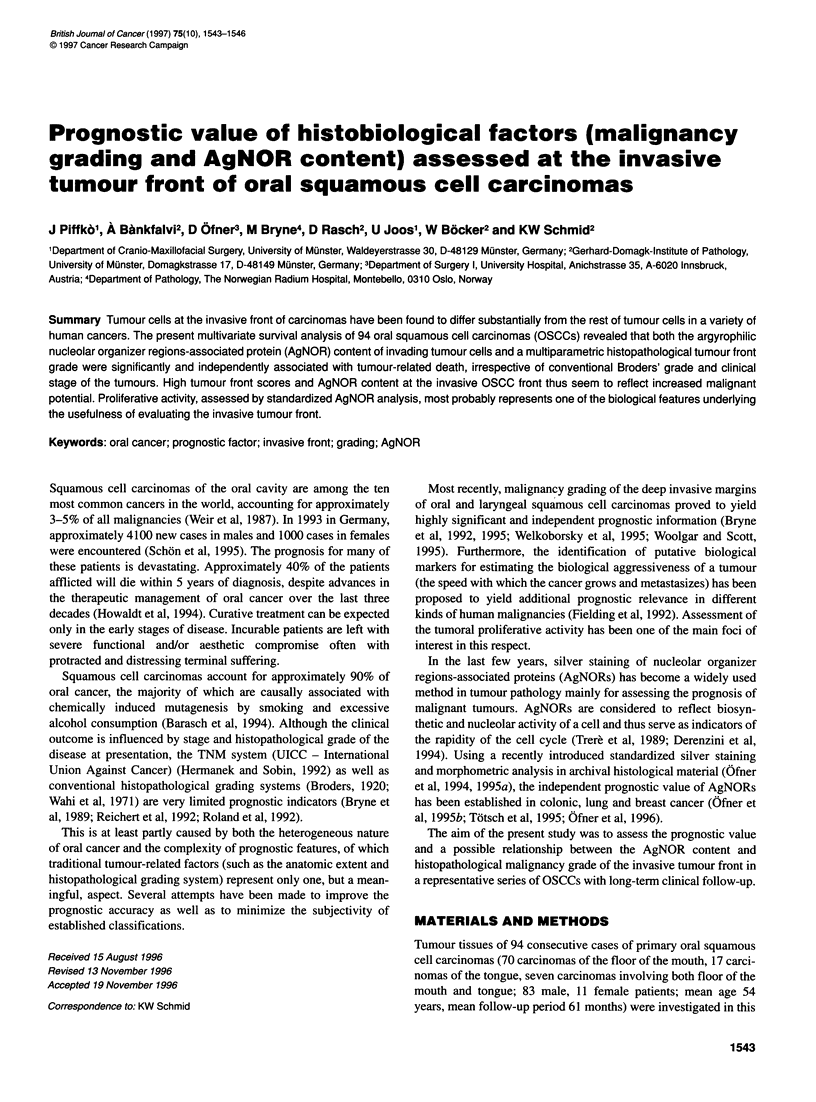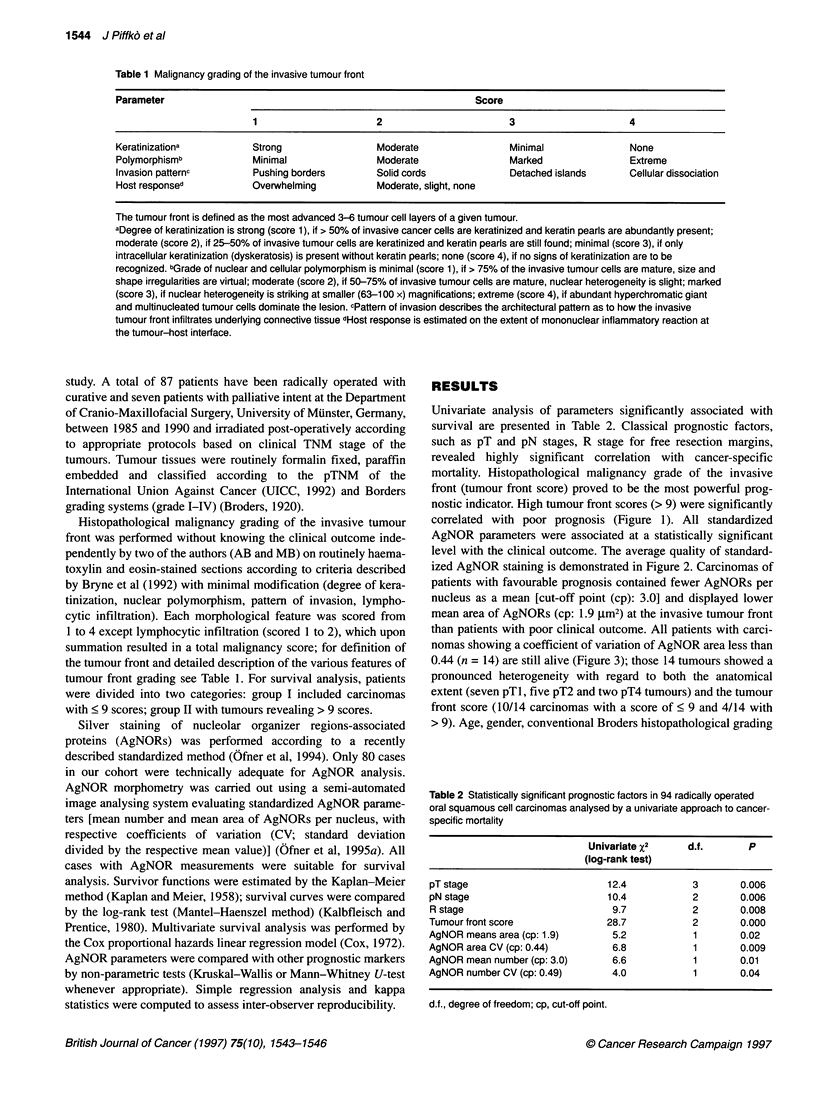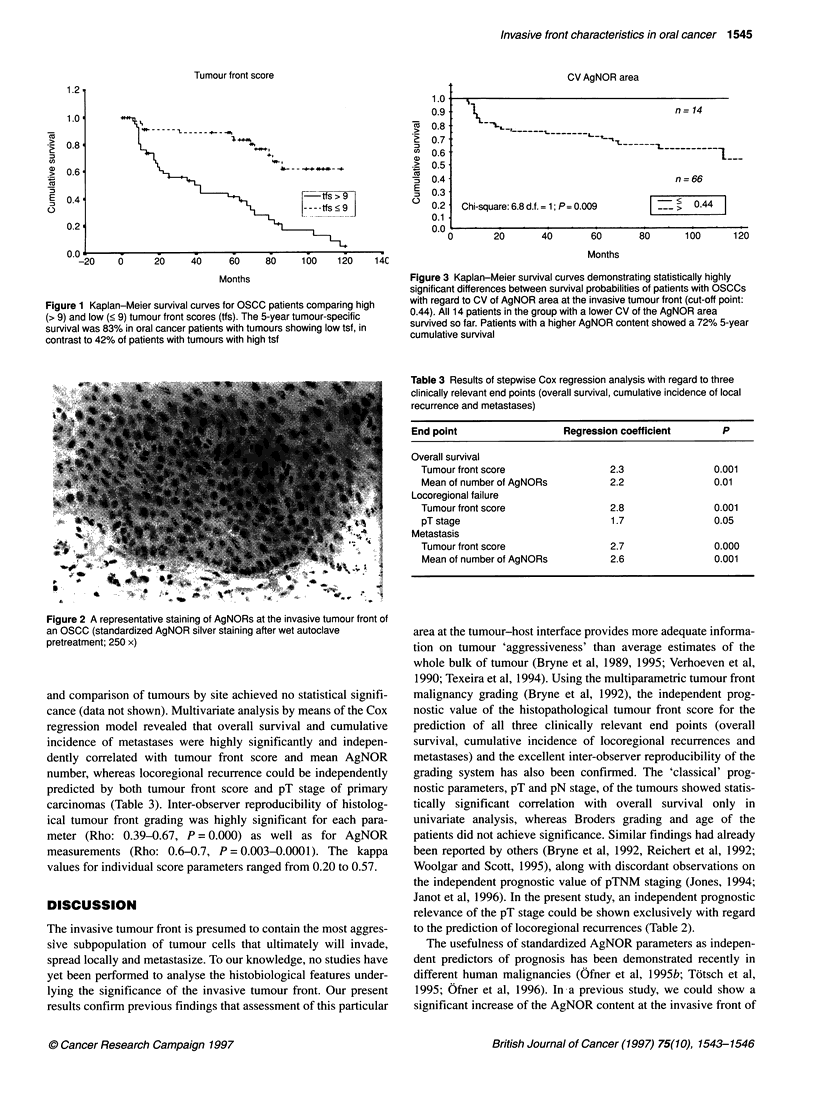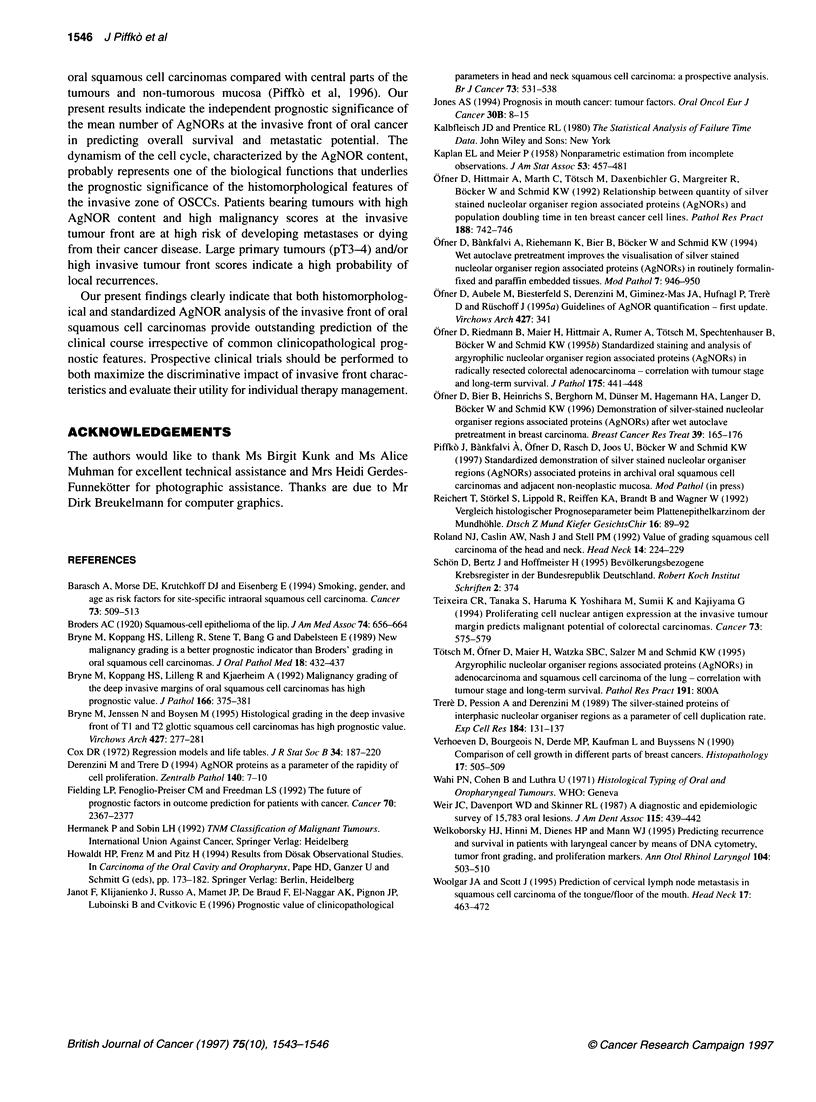# Prognostic value of histobiological factors (malignancy grading and AgNOR content) assessed at the invasive tumour front of oral squamous cell carcinomas.

**DOI:** 10.1038/bjc.1997.263

**Published:** 1997

**Authors:** J. PiffkÃ², A. BÃ nkfalvi, D. Ofner, M. Bryne, D. Rasch, U. Joos, W. BÃ¶cker, K. W. Schmid

**Affiliations:** Department of Cranio-Maxillofacial Surgery, University of MÃ¼nster, Germany.

## Abstract

**Images:**


British Journal of Cancer (1997) 75(10), 1543–1546
© 1997 Cancer Research Campaign

### Prognostic value of histobiological factors (malignancy grading and AgNOR content) assessed at the invasive tumour front of oral squamous cell carcinomas

**J Piffkò[1], À Bànkfalvi[2], D Öfner[3], M Bryne[4], D Rasch[2], U Joos[1], W Böcker[2] and KW Schmid[2]**

[1]Department of Cranio-Maxillofacial Surgery, University of Münster, Waldeyerstrasse 30, D-48129 Münster, Germany; [2]Gerhard-Domagk-Institute of Pathology, University of Münster, Domagkstrasse 17, D-48149 Münster, Germany; [3]Department of Surgery I, University Hospital, Anichstrasse 35, A-6020 Innsbruck, Austria; [4]Department of Pathology, The Norwegian Radium Hospital, Montebello, 0310 Oslo, Norway

**Summary** Tumour cells at the invasive front of carcinomas have been found to differ substantially from the rest of tumour cells in a variety of human cancers. The present multivariate survival analysis of 94 oral squamous cell carcinomas (OSCCs) revealed that both the argyrophilic nucleolar organizer regions-associated protein (AgNOR) content of invading tumour cells and a multiparametric histopathological tumour front grade were significantly and independently associated with tumour-related death, irrespective of conventional Broders' grade and clinical stage of the tumours. High tumour front scores and AgNOR content at the invasive OSCC front thus seem to reflect increased malignant potential. Proliferative activity, assessed by standardized AgNOR analysis, most probably represents one of the biological features underlying the usefulness of evaluating the invasive tumour front.

**Keywords**: oral cancer; prognostic factor; invasive front; grading; AgNOR

Squamous cell carcinomas of the oral cavity are among the ten most common cancers in the world, accounting for approximately 3–5% of all malignancies (Weir et al, 1987). In 1993 in Germany, approximately 4100 new cases in males and 1000 cases in females were encountered (Schön et al, 1995). The prognosis for many of these patients is devastating. Approximately 40% of the patients afflicted will die within 5 years of diagnosis, despite advances in the therapeutic management of oral cancer over the last three decades (Howaldt et al, 1994). Curative treatment can be expected only in the early stages of disease. Incurable patients are left with severe functional and/or aesthetic compromise often with protracted and distressing terminal suffering.

Squamous cell carcinomas account for approximately 90% of oral cancer, the majority of which are causally associated with chemically induced mutagenesis by smoking and excessive alcohol consumption (Barasch et al, 1994). Although the clinical outcome is influenced by stage and histopathological grade of the disease at presentation, the TNM system (UICC – International Union Against Cancer) (Hermanek and Sobin, 1992) as well as conventional histopathological grading systems (Broders, 1920; Wahi et al, 1971) are very limited prognostic indicators (Bryne et al, 1989; Reichert et al, 1992; Roland et al, 1992).

This is at least partly caused by both the heterogeneous nature of oral cancer and the complexity of prognostic features, of which traditional tumour-related factors (such as the anatomic extent and histopathological grading system) represent only one, but a meaningful, aspect. Several attempts have been made to improve the prognostic accuracy as well as to minimize the subjectivity of established classifications.

*Received 15 August 1996*
*Revised 13 November 1996*
*Accepted 19 November 1996*

*Correspondence to:* KW Schmid

Most recently, malignancy grading of the deep invasive margins of oral and laryngeal squamous cell carcinomas proved to yield highly significant and independent prognostic information (Bryne et al, 1992, 1995; Welkoborsky et al, 1995; Woolgar and Scott, 1995). Furthermore, the identification of putative biological markers for estimating the biological aggressiveness of a tumour (the speed with which the cancer grows and metastasizes) has been proposed to yield additional prognostic relevance in different kinds of human malignancies (Fielding et al, 1992). Assessment of the tumoral proliferative activity has been one of the main foci of interest in this respect.

In the last few years, silver staining of nucleolar organizer regions-associated proteins (AgNORs) has become a widely used method in tumour pathology mainly for assessing the prognosis of malignant tumours. AgNORs are considered to reflect biosynthetic and nucleolar activity of a cell and thus serve as indicators of the rapidity of the cell cycle (Trerè et al, 1989; Derenzini et al, 1994). Using a recently introduced standardized silver staining and morphometric analysis in archival histological material (Öfner et al, 1994, 1995*a*), the independent prognostic value of AgNORs has been established in colonic, lung and breast cancer (Öfner et al, 1995*b*; Tötsch et al, 1995; Öfner et al, 1996).

The aim of the present study was to assess the prognostic value and a possible relationship between the AgNOR content and histopathological malignancy grade of the invasive tumour front in a representative series of OSCCs with long-term clinical follow-up.

### MATERIALS AND METHODS

Tumour tissues of 94 consecutive cases of primary oral squamous cell carcinomas (70 carcinomas of the floor of the mouth, 17 carcinomas of the tongue, seven carcinomas involving both floor of the mouth and tongue; 83 male, 11 female patients; mean age 54 years, mean follow-up period 61 months) were investigated in this

**Table 1** Malignancy grading of the invasive tumour front

| Parameter | Score | | | |
|---|---|---|---|---|
| | 1 | 2 | 3 | 4 |
| Keratinization[a] | Strong | Moderate | Minimal | None |
| Polymorphism[b] | Minimal | Moderate | Marked | Extreme |
| Invasion pattern[c] | Pushing borders | Solid cords | Detached islands | Cellular dissociation |
| Host response[d] | Overwhelming | Moderate, slight, none | | |

The tumour front is defined as the most advanced 3–6 tumour cell layers of a given tumour.
[a]Degree of keratinization is strong (score 1), if > 50% of invasive cancer cells are keratinized and keratin pearls are abundantly present; moderate (score 2), if 25–50% of invasive tumour cells are keratinized and keratin pearls are still found; minimal (score 3), if only intracellular keratinization (dyskeratosis) is present without keratin pearls; none (score 4), if no signs of keratinization are to be recognized. [b]Grade of nuclear and cellular polymorphism is minimal (score 1), if > 75% of the invasive tumour cells are mature, size and shape irregularities are virtual; moderate (score 2), if 50–75% of invasive tumour cells are mature, nuclear heterogeneity is slight; marked (score 3), if nuclear heterogeneity is striking at smaller (63–100 ×) magnifications; extreme (score 4), if abundant hyperchromatic giant and multinucleated tumour cells dominate the lesion. [c]Pattern of invasion describes the architectural pattern as to how the invasive tumour front infiltrates underlying connective tissue [d]Host response is estimated on the extent of mononuclear inflammatory reaction at the tumour–host interface.

study. A total of 87 patients have been radically operated with curative and seven patients with palliative intent at the Department of Cranio-Maxillofacial Surgery, University of Münster, Germany, between 1985 and 1990 and irradiated post-operatively according to appropriate protocols based on clinical TNM stage of the tumours. Tumour tissues were routinely formalin fixed, paraffin embedded and classified according to the pTNM of the International Union Against Cancer (UICC, 1992) and Borders grading systems (grade I–IV) (Broders, 1920).

Histopathological malignancy grading of the invasive tumour front was performed without knowing the clinical outcome independently by two of the authors (AB and MB) on routinely haematoxylin and eosin-stained sections according to criteria described by Bryne et al (1992) with minimal modification (degree of keratinization, nuclear polymorphism, pattern of invasion, lymphocytic infiltration). Each morphological feature was scored from 1 to 4 except lymphocytic infiltration (scored 1 to 2), which upon summation resulted in a total malignancy score; for definition of the tumour front and detailed description of the various features of tumour front grading see Table 1. For survival analysis, patients were divided into two categories: group I included carcinomas with ≤ 9 scores; group II with tumours revealing > 9 scores.

Silver staining of nucleolar organizer regions-associated proteins (AgNORs) was performed according to a recently described standardized method (Öfner et al, 1994). Only 80 cases in our cohort were technically adequate for AgNOR analysis. AgNOR morphometry was carried out using a semi-automated image analysing system evaluating standardized AgNOR parameters [mean number and mean area of AgNORs per nucleus, with respective coefficients of variation (CV; standard deviation divided by the respective mean value)] (Öfner et al, 1995*a*). All cases with AgNOR measurements were suitable for survival analysis. Survivor functions were estimated by the Kaplan–Meier method (Kaplan and Meier, 1958); survival curves were compared by the log-rank test (Mantel–Haenszel method) (Kalbfleisch and Prentice, 1980). Multivariate survival analysis was performed by the Cox proportional hazards linear regression model (Cox, 1972). AgNOR parameters were compared with other prognostic markers by non-parametric tests (Kruskal–Wallis or Mann–Whitney *U*-test whenever appropriate). Simple regression analysis and kappa statistics were computed to assess inter-observer reproducibility.

### RESULTS

Univariate analysis of parameters significantly associated with survival are presented in Table 2. Classical prognostic factors, such as pT and pN stages, R stage for free resection margins, revealed highly significant correlation with cancer-specific mortality. Histopathological malignancy grade of the invasive front (tumour front score) proved to be the most powerful prognostic indicator. High tumour front scores (> 9) were significantly correlated with poor prognosis (Figure 1). All standardized AgNOR parameters were associated at a statistically significant level with the clinical outcome. The average quality of standardized AgNOR staining is demonstrated in Figure 2. Carcinomas of patients with favourable prognosis contained fewer AgNORs per nucleus as a mean [cut-off point (cp): 3.0] and displayed lower mean area of AgNORs (cp: 1.9 $\mu m^2$) at the invasive tumour front than patients with poor clinical outcome. All patients with carcinomas showing a coefficient of variation of AgNOR area less than 0.44 ($n$ = 14) are still alive (Figure 3); those 14 tumours showed a pronounced heterogeneity with regard to both the anatomical extent (seven pT1, five pT2 and two pT4 tumours) and the tumour front score (10/14 carcinomas with a score of ≤ 9 and 4/14 with > 9). Age, gender, conventional Broders histopathological grading

**Table 2** Statistically significant prognostic factors in 94 radically operated oral squamous cell carcinomas analysed by a univariate approach to cancer-specific mortality

| | Univariate $\chi^2$ (log-rank test) | d.f. | *P* |
|---|---|---|---|
| pT stage | 12.4 | 3 | 0.006 |
| pN stage | 10.4 | 2 | 0.006 |
| R stage | 9.7 | 2 | 0.008 |
| Tumour front score | 28.7 | 2 | 0.000 |
| AgNOR means area (cp: 1.9) | 5.2 | 1 | 0.02 |
| AgNOR area CV (cp: 0.44) | 6.8 | 1 | 0.009 |
| AgNOR mean number (cp: 3.0) | 6.6 | 1 | 0.01 |
| AgNOR number CV (cp: 0.49) | 4.0 | 1 | 0.04 |

d.f., degree of freedom; cp, cut-off point.

© Cancer Research Campaign 1997

**Figure 1** Kaplan–Meier survival curves for OSCC patients comparing high (> 9) and low (≤ 9) tumour front scores (tfs). The 5-year tumour-specific survival was 83% in oral cancer patients with tumours showing low tsf, in contrast to 42% of patients with tumours with high tsf

**Figure 2** A representative staining of AgNORs at the invasive tumour front of an OSCC (standardized AgNOR silver staining after wet autoclave pretreatment; 250 ×)

**Figure 3** Kaplan–Meier survival curves demonstrating statistically highly significant differences between survival probabilities of patients with OSCCs with regard to CV of AgNOR area at the invasive tumour front (cut-off point: 0.44). All 14 patients in the group with a lower CV of the AgNOR area survived so far. Patients with a higher AgNOR content showed a 72% 5-year cumulative survival

**Table 3** Results of stepwise Cox regression analysis with regard to three clinically relevant end points (overall survival, cumulative incidence of local recurrence and metastases)

| End point | Regression coefficient | *P* |
|---|---|---|
| Overall survival | | |
| Tumour front score | 2.3 | 0.001 |
| Mean of number of AgNORs | 2.2 | 0.01 |
| Locoregional failure | | |
| Tumour front score | 2.8 | 0.001 |
| pT stage | 1.7 | 0.05 |
| Metastasis | | |
| Tumour front score | 2.7 | 0.000 |
| Mean of number of AgNORs | 2.6 | 0.001 |

and comparison of tumours by site achieved no statistical significance (data not shown). Multivariate analysis by means of the Cox regression model revealed that overall survival and cumulative incidence of metastases were highly significantly and independently correlated with tumour front score and mean AgNOR number, whereas locoregional recurrence could be independently predicted by both tumour front score and pT stage of primary carcinomas (Table 3). Inter-observer reproducibility of histological tumour front grading was highly significant for each parameter (Rho: 0.39–0.67, $P = 0.000$) as well as for AgNOR measurements (Rho: 0.6–0.7, $P = 0.003–0.0001$). The kappa values for individual score parameters ranged from 0.20 to 0.57.

### DISCUSSION

The invasive tumour front is presumed to contain the most aggressive subpopulation of tumour cells that ultimately will invade, spread locally and metastasize. To our knowledge, no studies have yet been performed to analyse the histobiological features underlying the significance of the invasive tumour front. Our present results confirm previous findings that assessment of this particular area at the tumour–host interface provides more adequate information on tumour 'aggressiveness' than average estimates of the whole bulk of tumour (Bryne et al, 1989, 1995; Verhoeven et al, 1990; Texeira et al, 1994). Using the multiparametric tumour front malignancy grading (Bryne et al, 1992), the independent prognostic value of the histopathological tumour front score for the prediction of all three clinically relevant end points (overall survival, cumulative incidence of locoregional recurrences and metastases) and the excellent inter-observer reproducibility of the grading system has also been confirmed. The 'classical' prognostic parameters, pT and pN stage, of the tumours showed statistically significant correlation with overall survival only in univariate analysis, whereas Broders grading and age of the patients did not achieve significance. Similar findings had already been reported by others (Bryne et al, 1992, Reichert et al, 1992; Woolgar and Scott, 1995), along with discordant observations on the independent prognostic value of pTNM staging (Jones, 1994; Janot et al, 1996). In the present study, an independent prognostic relevance of the pT stage could be shown exclusively with regard to the prediction of locoregional recurrences (Table 2).

The usefulness of standardized AgNOR parameters as independent predictors of prognosis has been demonstrated recently in different human malignancies (Öfner et al, 1995*b*; Tötsch et al, 1995; Öfner et al, 1996). In a previous study, we could show a significant increase of the AgNOR content at the invasive front of

© Cancer Research Campaign 1997

oral squamous cell carcinomas compared with central parts of the tumours and non-tumorous mucosa (Piffkò et al, 1996). Our present results indicate the independent prognostic significance of the mean number of AgNORs at the invasive front of oral cancer in predicting overall survival and metastatic potential. The dynamism of the cell cycle, characterized by the AgNOR content, probably represents one of the biological functions that underlies the prognostic significance of the histomorphological features of the invasive zone of OSCCs. Patients bearing tumours with high AgNOR content and high malignancy scores at the invasive tumour front are at high risk of developing metastases or dying from their cancer disease. Large primary tumours (pT3–4) and/or high invasive tumour front scores indicate a high probability of local recurrences.

Our present findings clearly indicate that both histomorphological and standardized AgNOR analysis of the invasive front of oral squamous cell carcinomas provide outstanding prediction of the clinical course irrespective of common clinicopathological prognostic features. Prospective clinical trials should be performed to both maximize the discriminative impact of invasive front characteristics and evaluate their utility for individual therapy management.

### ACKNOWLEDGEMENTS

The authors would like to thank Ms Birgit Kunk and Ms Alice Muhman for excellent technical assistance and Mrs Heidi Gerdes-Funnekötter for photographic assistance. Thanks are due to Mr Dirk Breukelmann for computer graphics.

### REFERENCES

Barasch A, Morse DE, Krutchkoff DJ and Eisenberg E (1994) Smoking, gender, and age as risk factors for site-specific intraoral squamous cell carcinoma. *Cancer* **73**: 509–513

Broders AC (1920) Squamous-cell epithelioma of the lip. *J Am Med Assoc* **74**: 656–664

Bryne M, Koppang HS, Lilleng R, Stene T, Bang G and Dabelsteen E (1989) New malignancy grading is a better prognostic indicator than Broders' grading in oral squamous cell carcinomas. *J Oral Pathol Med* **18**: 432–437

Bryne M, Koppang HS, Lilleng R and Kjaerheim A (1992) Malignancy grading of the deep invasive margins of oral squamous cell carcinomas has high prognostic value. *J Pathol* **166**: 375–381

Bryne M, Jenssen N and Boysen M (1995) Histological grading in the deep invasive front of T1 and T2 glottic squamous cell carcinomas has high prognostic value. *Virchows Arch* **427**: 277–281

Cox DR (1972) Regression models and life tables. *J R Stat Soc B* **34**: 187–220

Derenzini M and Trere D (1994) AgNOR proteins as a parameter of the rapidity of cell proliferation. *Zentralb Pathol* **140**: 7–10

Fielding LP, Fenoglio-Preiser CM and Freedman LS (1992) The future of prognostic factors in outcome prediction for patients with cancer. *Cancer* **70**: 2367–2377

Hermanek P and Sobin LH (1992) *TNM Classification of Malignant Tumours*. International Union Against Cancer, Springer Verlag: Heidelberg

Howaldt HP, Frenz M and Pitz H (1994) Results from Dösak Observational Studies. In *Carcinoma of the Oral Cavity and Oropharynx*, Pape HD, Ganzer U and Schmitt G (eds), pp. 173–182. Springer Verlag: Berlin, Heidelberg

Janot F, Klijanienko J, Russo A, Mamet JP, De Braud F, El-Naggar AK, Pignon JP, Luboinski B and Cvitkovic E (1996) Prognostic value of clinicopathological parameters in head and neck squamous cell carcinoma: a prospective analysis. *Br J Cancer* **73**: 531–538

Jones AS (1994) Prognosis in mouth cancer: tumour factors. *Oral Oncol Eur J Cancer* **30B**: 8–15

Kalbfleisch JD and Prentice RL (1980) *The Statistical Analysis of Failure Time Data*. John Wiley and Sons: New York

Kaplan EL and Meier P (1958) Nonparametric estimation from incomplete observations. *J Am Stat Assoc* **53**: 457–481

Öfner D, Hittmair A, Marth C, Tötsch M, Daxenbichler G, Margreiter R, Böcker W and Schmid KW (1992) Relationship between quantity of silver stained nucleolar organiser region associated proteins (AgNORs) and population doubling time in ten breast cancer cell lines. *Pathol Res Pract* **188**: 742–746

Öfner D, Bànkfalvi A, Riehemann K, Bier B, Böcker W and Schmid KW (1994) Wet autoclave pretreatment improves the visualisation of silver stained nucleolar organiser region associated proteins (AgNORs) in routinely formalin-fixed and paraffin embedded tissues. *Mod Pathol* **7**: 946–950

Öfner D, Aubele M, Biesterfeld S, Derenzini M, Giminez-Mas JA, Hufnagl P, Trerè D and Rüschoff J (1995*a*) Guidelines of AgNOR quantification – first update. *Virchows Arch* **427**: 341

Öfner D, Riedmann B, Maier H, Hittmair A, Rumer A, Tötsch M, Spechtenhauser B, Böcker W and Schmid KW (1995*b*) Standardized staining and analysis of argyrophilic nucleolar organiser region associated proteins (AgNORs) in radically resected colorectal adenocarcinoma – correlation with tumour stage and long-term survival. *J Pathol* **175**: 441–448

Öfner D, Bier B, Heinrichs S, Berghorn M, Dünser M, Hagemann HA, Langer D, Böcker W and Schmid KW (1996) Demonstration of silver-stained nucleolar organiser regions associated proteins (AgNORs) after wet autoclave pretreatment in breast carcinoma. *Breast Cancer Res Treat* **39**: 165–176

Piffkò J, Bànkfalvi À, Öfner D, Rasch D, Joos U, Böcker W and Schmid KW (1997) Standardized demonstration of silver stained nucleolar organiser regions (AgNORs) associated proteins in archival oral squamous cell carcinomas and adjacent non-neoplastic mucosa. *Mod Pathol* (in press)

Reichert T, Störkel S, Lippold R, Reiffen KA, Brandt B and Wagner W (1992) Vergleich histologischer Prognoseparameter beim Plattenepithelkarzinom der Mundhöhle. *Dtsch Z Mund Kiefer GesichtsChir* **16**: 89–92

Roland NJ, Caslin AW, Nash J and Stell PM (1992) Value of grading squamous cell carcinoma of the head and neck. *Head Neck* **14**: 224–229

Schön D, Bertz J and Hoffmeister H (1995) Bevölkerungsbezogene Krebsregister in der Bundesrepublik Deutschland. *Robert Koch Institut Schriften* **2**: 374

Teixeira CR, Tanaka S, Haruma K Yoshihara M, Sumii K and Kajiyama G (1994) Proliferating cell nuclear antigen expression at the invasive tumour margin predicts malignant potential of colorectal carcinomas. *Cancer* **73**: 575–579

Tötsch M, Öfner D, Maier H, Watzka SBC, Salzer M and Schmid KW (1995) Argyrophilic nucleolar organiser regions associated proteins (AgNORs) in adenocarcinoma and squamous cell carcinoma of the lung – correlation with tumour stage and long-term survival. *Pathol Res Pract* **191**: 800A

Trerè D, Pession A and Derenzini M (1989) The silver-stained proteins of interphasic nucleolar organiser regions as a parameter of cell duplication rate. *Exp Cell Res* **184**: 131–137

Verhoeven D, Bourgeois N, Derde MP, Kaufman L and Buyssens N (1990) Comparison of cell growth in different parts of breast cancers. *Histopathology* **17**: 505–509

Wahi PN, Cohen B and Luthra U (1971) *Histological Typing of Oral and Oropharyngeal Tumours*. WHO: Geneva

Weir JC, Davenport WD and Skinner RL (1987) A diagnostic and epidemiologic survey of 15,783 oral lesions. *J Am Dent Assoc* **115**: 439–442

Welkoborsky HJ, Hinni M, Dienes HP and Mann WJ (1995) Predicting recurrence and survival in patients with laryngeal cancer by means of DNA cytometry, tumor front grading, and proliferation markers. *Ann Otol Rhinol Laryngol* **104**: 503–510

Woolgar JA and Scott J (1995) Prediction of cervical lymph node metastasis in squamous cell carcinoma of the tongue/floor of the mouth. *Head Neck* **17**: 463–472

© Cancer Research Campaign 1997